# Measles Epidemics in Romania: Lessons for Public Health and Future Policy

**DOI:** 10.3389/fpubh.2019.00098

**Published:** 2019-04-25

**Authors:** Stefan Dascalu

**Affiliations:** ^1^Department of Zoology, University of Oxford, Oxford, United Kingdom; ^2^Avian Influenza Virus, Pirbright Institute (BBSRC), Woking, United Kingdom

**Keywords:** Romania, measles, epidemic, public health, vaccination, outbreak

## Abstract

Measles is a highly infectious viral disease that continues to be a challenge for many countries worldwide. Although significant improvements have been observed since the introduction of vaccines, measles remains endemic in Romania. Contributing factors include vaccine hesitancy, difficulties in delivering doses to the population, and even the lack of sufficient vaccine supplies. These problems are further exacerbated by an inadequate implementation of public health measures, ranging from inefficient communication programs to the absence of a legislative framework concerning immunization. Moreover, many of the recent outbreaks were associated with chains of transmission in other countries, thus making the control of measles in Romania relevant at an international level. As many difficulties exist, understanding the key factors that limit the success of public health programs may provide guidance in shaping future strategies. Because similar issues are being faced in various other countries, the management of measles in Romania offers valuable lessons for researchers and policy-makers alike.

## Introduction

Measles is a highly infectious viral disease which can result in severe complications and even death (1, 2). To this day, measles remains one of the leading causes of childhood mortality worldwide, with most of the deaths occurring in individuals younger than 5 years of age (1). Nevertheless, a cheap and safe vaccine exists, and constant international efforts have been targeted toward eliminating the disease (3). However, for this to be achieved, a very high vaccination coverage (>95%) needs to be achieved and maintained for extensive periods of time.

**Figure 1:**
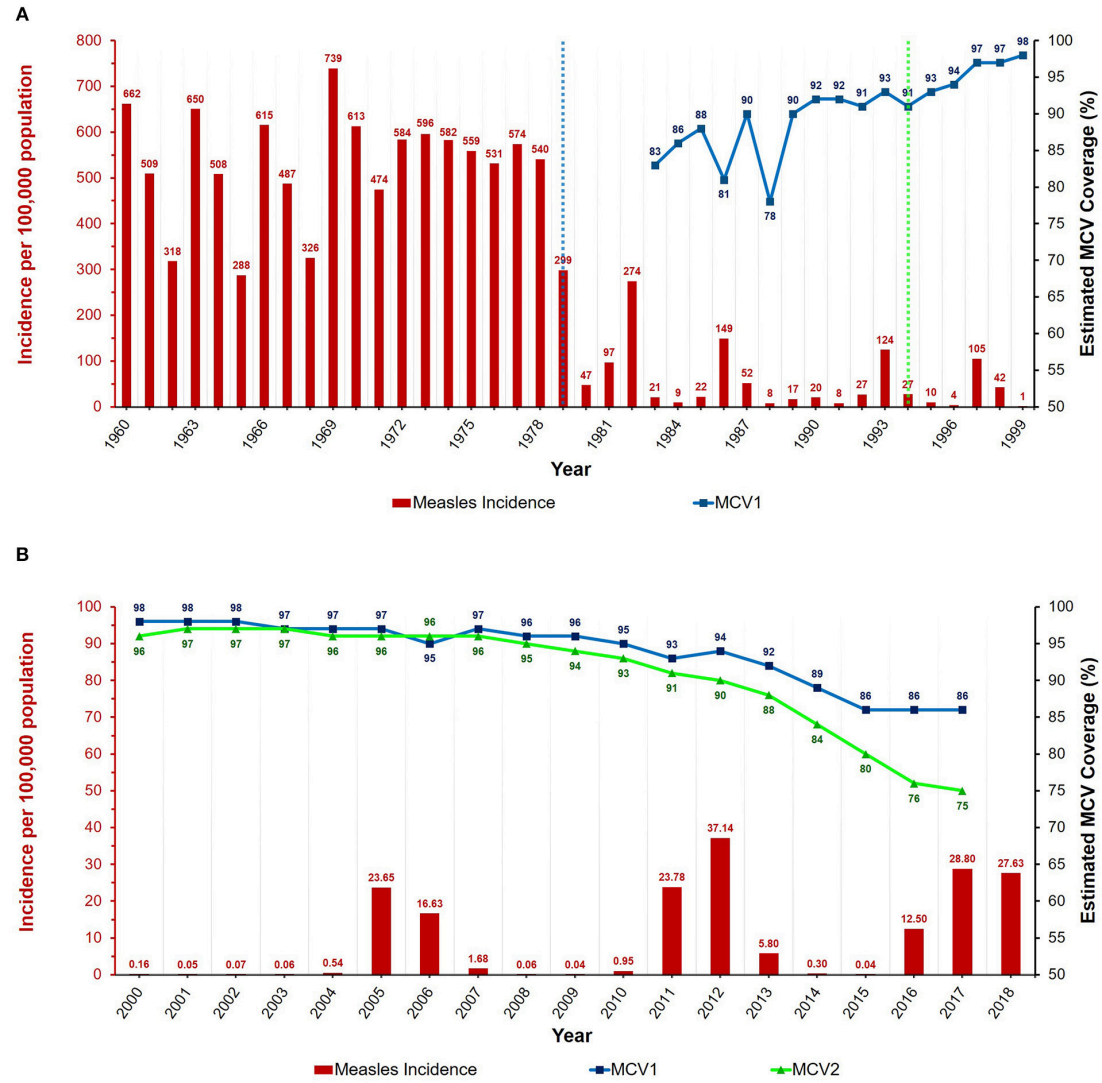
Annual measles incidence and MCV coverage in Romania from **(A)** 1960 to 1999 and **(B)** 2000 to 2018. Blue and green dotted lines represent the introduction of the MCV1 (1979) and MCV2 (1994) vaccines, respectively. Data available in Supplementary Tables 1, 2. Estimates for MCV1 coverage are only from 1983 onwards, and MCV2 coverage estimates are unavailable before the year 2000. Coverage data for 2018 unavailable at the time of writing.

In Romania, the first dose of the monovalent measles-containing vaccine (MCV1) was introduced in 1979 for children aged 9–11 months, and the second dose (MCV2) was implemented in 1994 for children 6–7 years of age (Figure 1A) (2). The replacement of the first dose by the trivalent measles, mumps and rubella (MMR) vaccine occurred in 2004, with the recommended age of inoculation being 12–15 months. The second dose was scheduled as part of school-based vaccination programs, and was aimed at children aged 6–7 years (2). In 2015, the standard age for inoculation with the second dose of the MMR vaccine was lowered to 5 years of age and the vaccine's delivery was moved to health centers instead of schools (2, 4).

Although these public health strategies have achieved and maintained a high vaccination coverage, outbreaks of variable severity have still been taking place, sometimes exhibiting elevated levels of mortality, especially in young children (2). Moreover, most of the recent epidemics were linked with outbreaks in other countries, thus making the outcomes of measles control in Romania relevant on an international scale (5–9). In many of these situations, the transmission of the virus was further facilitated by an atypical onset of symptoms and serious clinical complications (6). Cumulatively, these factors delayed accurate diagnosis and appropriate containment, thereby allowing measles to spread at a faster rate than the authorities could provide efficient intervention and control.

In this paper, a synthesis of the available information about measles in Romania is provided. Recent outbreaks, some of their underlying factors, and specific control measures are presented in order to illustrate the successes and failures of national public health strategies. At the same time, possible solutions are assessed in terms of their potential to limit the impact of future epidemics.

## Outbreaks and Trends in Annual Incidence

After the introduction of the measles-containing vaccine in 1979, the dynamics of the disease changed, exhibiting a decreased annual incidence and longer inter-epidemic periods (see Figure 1A) (10–17). As opposed to the pre-vaccine era, when the average yearly incidence (per 100,000 population) exceeded 500, the magnitude of this value was almost 10 times lower from 1980 to 1999 (17, 18). Furthermore, the incidence also underwent an overall decrease due to mass vaccination campaigns and the intervention measures carried out in response to epidemics, thereby reaching very low values by the year 2000 (10–18). As such, the most notable outbreaks of the twentieth century were the ones with peak incidences in 1982, 1986, 1993, and 1997.

Although the disease burden caused by measles had decreased substantially in Romania by the beginning of the current millennium, the epidemics of 2004–2007 and 2011–2013 deserve attention, as well as the one that began in 2016 (Figure 1B). Generally, the genotypes associated with endemic transmission in Romania are D4 and D5, and these were indeed responsible for most of the epidemics of the current century (2, 19–21). For example, the D4 variant was dominant during the large epidemic of December 2004 to early 2007. This genotype became endemic to Romania, and was also the main variant detected during the subsequent epidemic of 2011–2013 (21). In contrast, B3 was the main genotype associated with the 2016 outbreak (2). This variant had never been linked with endemic circulation in Romania and had only rarely been detected in Europe previously (20, 22, 23). However, in <1 year, several European countries reported outbreaks involving the B3 genotype, with many cases being linked with the Romanian epidemic (2, 6, 7, 9, 24–28).

**Figure 2:**
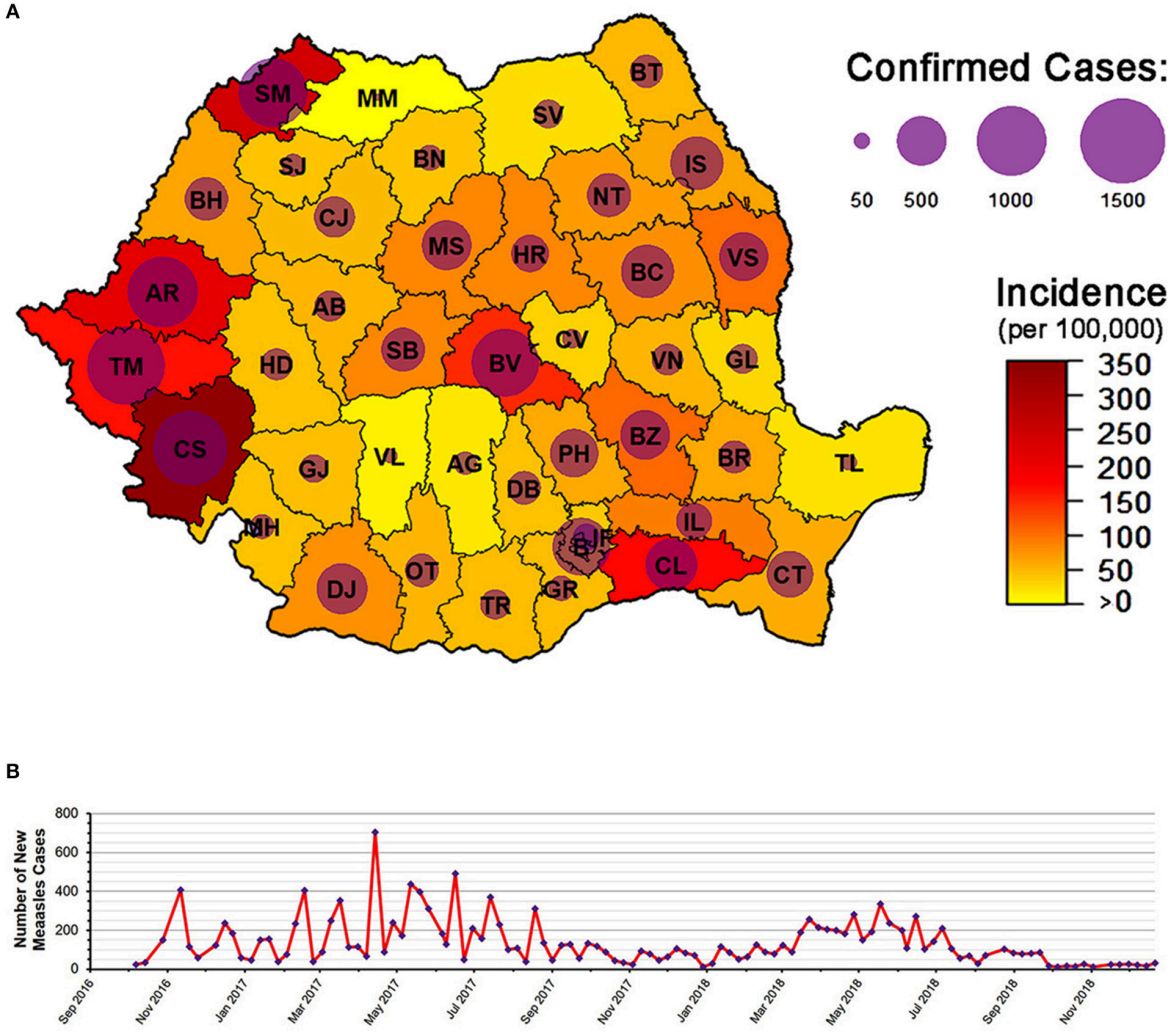
The 2016 Romanian measles epidemic. **(A)** The epidemic as of 21 December 2018: 15,587 total number of cases, with 59 confirmed deaths. County codes are depicted in the figure. **(B)** Number of new measles cases from October 2016 to December 2018. Figure based on data from the weekly reports released by the Romanian National Center for the Surveillance and Control of Communicable Diseases (CNSCBT). For the evolution of the epidemic by county, see Supplementary Video. Epidemic data (at national level) is available in Supplementary Table 3.

The measles outbreak of 2016 resulted in more than 15,500 cases with a total of 59 deaths by the end of 2018 (Figure 2) (29). This high mortality was mostly in individuals that suffered from pre-existing medical conditions, very few of them having been vaccinated against measles (30). Furthermore, many of these deaths were in children below the age when efficient rates of seroconversion exist, and thus vaccination would not have been recommended (31). The 2016 outbreak was also considered to pose a major risk concerning the export of measles, and several cases in Europe were indeed traced back to Romania (2, 6–9). As such, the primary cases of some recent outbreaks in Ireland, Greece, Bulgaria, and Belgium were either of Romanian nationality or had traveled to Romania before becoming infectious. Therefore, it is important to note that Romania poses an increased risk of acting as a reservoir for measles, having the potential to seed epidemics on an international scale.

## Vaccination

As no specific treatment for measles exists, vaccination remains the only effective measure in combating this disease (1, 2). In Romania, both of the MCV doses are required to be administered by trained medical personnel. Additionally, since 2011, legislation exists that obliges all family doctors and healthcare professionals involved in the act of immunization to periodically report on the vaccination status of children (32). On a yearly basis, the coverage was estimated to be higher than 95% for both MCV1 and MCV2 vaccines in the early 2000s, but a declining trend has been observed after 2010 (see Figure 2) (2, 17, 33). Therefore, from an estimated coverage of >96% with the first dose of the vaccine, the values declined to ~89% in 2014, and ~86% after 2015. Moreover, the second dose achieved an estimated coverage of <80% after 2015 compared to >95% in the early 2000s. In many cases, the targeted vaccination coverage for measles (i.e., >95%) was unable to be reached due to several factors, such as the active refusal of parents to vaccinate their children or even the lack of sufficient vaccine supplies (2, 10, 21). For example, the monthly coverage of the first scheduled dose of the measles vaccine was under 90% in 2009, reaching staggering lows of ~53% in November and 43% in December (10). This was mostly the result of an insufficient national stock of vaccines, and thus there is an impending need for the authorities to ensure that enough doses are available to the population, especially in the case of an ongoing measles outbreak.

In response to measles epidemics, additional vaccination campaigns have been conducted over the years to supplement the national program (2, 12, 13). For example, a mass immunization was carried out from 1998 to 1999 as a measure of controlling the then ongoing outbreak (13). Likewise, from 2005 to 2006, a supplementary monovalent inoculation was recommended to children between 7 months and 7 years of age. Furthermore, in response to the measles outbreak of 2011, an extra dose of the vaccine was targeted at children in the same age category and resulted in more than 4,500 individuals being immunized, irrespective of prior vaccination status (2, 11). Similarly, in 2016, one of the measures used to control the spread of the measles epidemic was a catch-up vaccination campaign aimed at children belonging to various age groups (34). However, variable degrees of success were observed. For example, by the end of May 2017, a high vaccination coverage with the first dose (98.9%) was obtained in children 1–4 years of age in the Timis county. In contrast, only 62.5% of children aged 5–9 years were immunized with the first dose, and only 39.1% received the second inoculation. Moreover, in Caras-Severin (then one of the most affected counties), a mere 31.4% of children aged 1–4 years were vaccinated with the first dose and only 15.3 and 17.6% of children aged 5–9 years received the first and second vaccines, respectively. Therefore, the delivery of the doses to the population remains an important issue, particularly during epidemics.

An additional problem is represented by the low vaccine uptake in vulnerable communities, and more targeted measures would be needed to achieve good immunization coverage in these populations (20, 21, 35). Moreover, the difficulties in communicating with and offering healthcare to vulnerable subsets of the population further contributed to recent outbreaks at both the national and international levels. For example, members of the Roma and Sinti minorities were involved in the transmission of the Romanian measles strain in at least 10 other European countries from 2005 to 2007 (20). Similarly, from 2016 onwards, many transmission chains in Europe involved members of these minority groups (6, 8, 28). With this in mind, if public health strategies were to take into account specific socio-cultural factors and be directed in a more targeted manner toward vulnerable communities, the severity of future outbreaks would probably be diminished (35).

The declining trends in vaccination coverage in the context of the recent measles epidemics have also constituted the basis for recurrent discussions about mandatory immunization (36–38). Although national vaccination strategies do exist in Romania, a legislative framework that states and enforces the exact responsibilities of and benefits for the concerned parties has never been employed. As such, even if the government provides the vaccines which are part of the national immunization program (including the MMR) free of charge, parents can still opt out without having to legally justify this action. Furthermore, although Romanian medical practitioners receive a small financial remuneration from the government for each vaccination consult, this has been regarded by many as insufficient, and may therefore not provide enough motivation to achieve high coverage rates (39, 40). With these factors in mind, examples do exist of countries where vaccination coverage has been increased by a more explicit form of legal enforcement (e.g., school entry laws or substantial financial incentives/penalties) (41). However, because individual freedoms are usually concerned, it is important to emphasize that such measures might not be viewed favorably by the public. Therefore, if the social, economic, and cultural factors are not evaluated appropriately, a legal enforcement of vaccination may lead to public unrest and the inability to deliver the desired outcomes.

## Public Awareness and Views on Immunization

Medical practitioners are offered regular training sessions by the county branches of the Romanian National Institute of Public Health (INSP), which are aimed at providing them with skills in vaccine delivery and in communicating with patients (4). Furthermore, in the case of an ongoing epidemic, strategies exist for disseminating information to the community (42). For example, during the 2016 outbreak, advertisements with widely-known public figures, such as actors and musicians were aired on television, presenting information about vaccine-preventable diseases and their associated burdens (43, 44). Similarly, the Romanian Ministry of Health also developed an official website that presents accessible information about immunization and explains why achieving a high vaccination coverage is beneficial for society as a whole (43). Additionally, campaigns were carried out in which posters, leaflets and other informative materials were provided to healthcare practitioners and the general population (2).

Overall, the majority of the public (83%) believes that immunizing children as part of the national vaccination strategy is beneficial, according to a social study carried out in 2017 (45). Furthermore, 87% of the surveyed individuals considered that the benefits of vaccination far outweigh potential side-effects. However, despite the overall positive views on immunization and possibly because of the inefficient implementation of public health measures, the dangers associated with vaccine-preventable diseases, such as measles remain an important problem. One key element that potentially adds to the ineffectiveness of measles control in Romania is the belief that the healthcare system is unable to provide efficient services. As such, 40% of individuals consider that this is a direct cause of the frequent changes of health ministers, while another 40% believe that the recent national shortage of vaccine supplies is due to the Ministry of Health being unable to handle the situation (45). Correspondingly, another study concerning cancer treatment in Romania revealed that only about a third of the surveyed persons had a high trust in the effectiveness of state-funded healthcare facilities (38). The individuals placed corruption, insufficient staffing, and the inadequate supply of medication among the top five problems of the national healthcare system. Cumulatively, these perceptions may impact the effectiveness of all public health programs in Romania, including the management and control of vaccine-preventable diseases.

Religious arguments have also been used against vaccination on many occasions. According to a 2011 census, more than 85% of the population identified as Orthodox Christian, and this factor is likely to influence the views of the public concerning immunization (46). The official stance of the Romanian Orthodox Church is that it supports the use of vaccines if they are not destined for commercial purposes and if individual freedoms are respected (47). Additionally, a collaboration protocol has been established between the government and the Orthodox Church in 2008 in order to provide “*spiritual and medical assistance*” to the public, including access to and information about vaccines (47–49). However, the Church often ignores the association of its name with most events or campaigns that advocate against immunization, and many religious officials actively encourage parents to refuse the vaccination of their children (47, 50, 51).

The global anti-vaccine movement has also become more prevalent in Romania in recent years (21, 38). The increase in the number of books, websites, social media pages and even public appearances of individuals that are against vaccination are a reflection of the same phenomenon occurring on an international scale (52). Moreover, the media coverage of anti-vaccine advocates further enhances public mistrust and detrimental attitudes toward healthcare officials who are involved in immunization activities (38, 53, 54). Together, these negative views can also affect the degree to which parents report measles to healthcare providers, thus further contributing to the spread of infectious diseases. With these factors in mind, the analysis of anti-vaccine blogs and other types of social media coupled with conducting surveys that inquire about the issues of vaccination might prove to be extremely useful when designing national public health campaigns (55). Furthermore, assessing the main factors that are presented as arguments against vaccination would allow for more efficient strategies concerning both immunizations and raising awareness. This information may also facilitate the identification of specific subsets of the population, thus enabling more targeted measures to be designed for maximizing the efficiency of national public health programs.

## Conclusions

There are a multitude of factors contributing to the negative outcomes of the prevention and control of measles in Romania, such as difficulties in vaccine delivery, the public's views on immunization, and even the absence of a legislative framework regarding vaccination. Consequently, although an elaborate methodology exists, most of the outbreaks that have occurred in recent years often failed to be contained in an appropriate way. With this in mind, the outcomes of public health measures in Romania will strictly depend on the way in which current and future policies will be implemented. The results will be contingent on thorough evaluations of existing methodologies, efficient public awareness campaigns, adequate intervention strategies, and more targeted measures toward vulnerable subsets of the population. Regarding this latter factor, conducting community-level serosurveys might have the potential to identify individuals that are at high risk of infection with measles. At the same time, understanding why people are hesitant about, or actively opposed to vaccination may provide invaluable insights for designing tactics to address these problems. Currently, it remains to be seen how Romanian politicians, public health authorities, and members of the civil society will address these issues in the upcoming years. Because similar problems are being faced on an international scale, the lessons learned from the measles epidemics in Romania may provide guidance in shaping future strategies not only for the Romanian authorities, but also for other countries that are challenged by this disease.

## Author Contributions

The author confirms being the sole contributor of this work and has approved it for publication.

### Conflict of Interest Statement

The author declares that the research was conducted in the absence of any commercial or financial relationships that could be construed as a potential conflict of interest.

## Supplementary Material

Supplementary Table 1 Measles incidence and MCV coverage estimates for Romania 1960–2018.

Supplementary Table 2 The 2016 Romanian measles epidemic: incidence and total number of cases at 23 December 2018.

Supplementary Table 3 The evolution of the 2016 measles epidemic in Romania.

Supplementary Video 1 The evolution of the 2016 Romanian measles epidemic.
