# Supplementary material for: Measles Epidemics in Romania: Lessons for Public Health and Future Policy
Source: Front Public Health. 2019 Apr 25;7:98. doi: 10.3389/fpubh.2019.00098 (PMC6496956; doi:10.3389/fpubh.2019.00098)
Supplement: Supplementary Table 1 — Measles incidence and MCV coverage estimates for Romania 1960–2018. [file Table_1.DOCX]

**Supplementary Table 1:** *Measles incidence and MCV coverage estimates for Romania 1960-2018*

| **Year** | **Population Size**^1^ | **Number of Cases^2^** | **Incidence** | **Vaccination Coverage Estimates^3^** | |
| --- | --- | --- | --- | --- | --- |
|  |  |  |  | **MCV1** | **MCV2** |
| 1960 | 18,403,414 | 121831 | 662 | N/A | N/A |
| 1961 | 18,566,932 | 94533 | 509 | N/A | N/A |
| 1962 | 18,680,721 | 59382 | 318 | N/A | N/A |
| 1963 | 18,813,131 | 122374 | 650 | N/A | N/A |
| 1964 | 18,927,081 | 96170 | 508 | N/A | N/A |
| 1965 | 19,027,367 | 54707 | 288 | N/A | N/A |
| 1966 | 19,140,783 | 117801 | 615 | N/A | N/A |
| 1967 | 19,284,814 | 93931 | 487 | N/A | N/A |
| 1968 | 19,720,984 | 64200 | 326 | N/A | N/A |
| 1969 | 20,010,178 | 147859 | 739 | N/A | N/A |
| 1970 | 20,252,541 | 124060 | 613 | N/A | N/A |
| 1971 | 20,469,658 | 97084 | 474 | N/A | N/A |
| 1972 | 20,662,648 | 120602 | 584 | N/A | N/A |
| 1973 | 20,827,525 | 124057 | 596 | N/A | N/A |
| 1974 | 21,028,841 | 122470 | 582 | N/A | N/A |
| 1975 | 21,245,103 | 118703 | 559 | N/A | N/A |
| 1976 | 21,445,698 | 113907 | 531 | N/A | N/A |
| 1977 | 21,657,569 | 124227 | 574 | N/A | N/A |
| 1978 | 21,854,622 | 118124 | 540 | N/A | N/A |
| 1979 | 22,048,305 | 65820 | 299 | N/A | N/A |
| 1980 | 22,201,387 | 10476 | 47 | N/A | N/A |
| 1981 | 22,352,635 | 21584 | 97 | N/A | N/A |
| 1982 | 22,477,703 | 61682 | 274 | N/A | N/A |
| 1983 | 22,553,074 | 4723 | 21 | 83 | N/A |
| 1984 | 22,624,505 | 2108 | 9 | 86 | N/A |
| 1985 | 22,724,836 | 5007 | 22 | 88 | N/A |
| 1986 | 22,823,479 | 34037 | 149 | 81 | N/A |
| 1987 | 22,940,430 | 11833 | 52 | 90 | N/A |
| 1988 | 23,053,552 | 1810 | 8 | 78 | N/A |
| 1989 | 23,151,564 | 3870 | 17 | 90 | N/A |
| 1990 | 23,206,720 | 4690 | 20 | 92 | N/A |
| 1991 | 23,185,084 | 1773 | 8 | 92 | N/A |
| 1992 | 22,788,969 | 6061 | 27 | 91 | N/A |
| 1993 | 22,755,260 | 28321 | 124 | 93 | N/A |
| 1994 | 22,730,622 | 6228 | 27 | 91 | N/A |
| 1995 | 22,680,951 | 2188 | 10 | 93 | N/A |
| 1996 | 22,607,620 | 906 | 4 | 94 | N/A |
| 1997 | 22,545,925 | 23579 | 105 | 97 | N/A |
| 1998 | 22,502,803 | 9547 | 42 | 97 | N/A |
| 1999 | 22,458,022 | 240 | 1 | 98 | N/A |
| 2000 | 22,435,205 | 35 | 0.156 | 98 | 96 |
| 2001 | 22,408,393 | 11 | 0.049 | 98 | 97 |
| 2002 | 21,675,775 | 15 | 0.069 | 98 | 97 |
| 2003 | 21,574,365 | 12 | 0.056 | 97 | 97 |
| 2004 | 21,451,845 | 116 | 0.541 | 97 | 96 |
| 2005 | 21,319,673 | 5043 | 23.654 | 97 | 96 |
| 2006 | 21,193,749 | 3524 | 16.628 | 95 | 96 |
| 2007 | 20,882,980 | 350 | 1.676 | 97 | 96 |
| 2008 | 20,537,848 | 12 | 0.058 | 96 | 95 |
| 2009 | 20,367,437 | 8 | 0.039 | 96 | 94 |
| 2010 | 20,246,798 | 193 | 0.953 | 95 | 93 |
| 2011 | 20,121,641 | 4784 | 23.775 | 93 | 91 |
| 2012 | 20,060,182 | 7450 | 37.138 | 94 | 90 |
| 2013 | 19,988,694 | 1159 | 5.798 | 92 | 88 |
| 2014 | 19,913,193 | 59 | 0.296 | 89 | 84 |
| 2015 | 19,819,477 | 7 | 0.035 | 86 | 80 |
| 2016 | 19,473,624 | 2435 | 12.504 | 86 | 76 |
| 2017 | 19,310,216 | 5562 | 28.803 | 86 | 75 |
| 2018 | 19,213,665* | 5308 | 27.626 | N/A | N/A |

**^1^** Data obtained from the National Institute of Statistics. *Statistical Yearbook of 2016*. Available online: [http://www.insse.ro/cms/sites/default/files/field/publicatii/anuar_statistic_al_romaniei_2016_format_carte.pdf](http://www.insse.ro/cms/sites/default/files/field/publicatii/anuar_statistic_al_romaniei_2016_format_carte.pdf%20) (accessed on 18 February 2019)

**^2^** Data obtained from the Romanian National Centre for the Surveillance and Control of Communicable Diseases (CNSCBT). Available online: <http://insulaindoielii.ro/wp-content/uploads/2017/06/cazuri_si_acoperire_vaccinala.pdf?fbclid=IwAR3rxe62W0VyqbMdhIxVeCdhc3HNfH9JTA1QVFIRzdqrX6Ie3RtHH4pJGSc> (accessed on 18 February 2019)

**^3^** Data obtained from *WHO/UNICEF estimates of national immunization coverage*. World Health Organization; Available online[: https://www.who.int/immunization/monitoring_surveillance/routine/coverage/en/index4.html](file:///E:\BK\Partitia%20D\Documents\Old%20(undergrad)\Measles%20Article%20Romania\2018_last\Submission%20Clujul%20Medical\%20https\www.who.int\immunization\monitoring_surveillance\routine\coverage\en\index4.html) (accessed on 18 February 2019)

* Estimate calculated by the population projection obtained from: United Nations, Department of Economic and Social Affairs, Population Division (2017). World Population Prospects: The 2017 Revision. Available online: <https://population.un.org/wpp/> (accessed on 18 February 2019)
