# Supplementary material for: Measles Epidemics in Romania: Lessons for Public Health and Future Policy
Source: Front Public Health. 2019 Apr 25;7:98. doi: 10.3389/fpubh.2019.00098 (PMC6496956; doi:10.3389/fpubh.2019.00098)
Supplement: Supplementary Table 2 — The 2016 Romanian measles epidemic: incidence and total number of cases at 23 December 2018. [file Table_2.DOCX]

**Supplementary Table 2:** *The 2016 Romanian measles epidemic: incidence and total number of cases at 23 December 2018.*

| **County** | **County Code** | **Population^4^** | **Number of Confirmed Measles Cases^5^** | **Incidence** |
| --- | --- | --- | --- | --- |
|  |  |  | **21-Dec-18** | **21-Dec-18** |
| Alba | AB | 380,976 | 191 | 50.13 |
| Arad | AR | 473,946 | 1015 | 214.16 |
| Arges | AG | 646,333 | 105 | 16.25 |
| Bacau | BC | 746,566 | 572 | 76.62 |
| Bihor | BH | 619,102 | 380 | 61.38 |
| Bistrita-Nasaud | BN | 329,188 | 131 | 39.79 |
| Botosani | BT | 455,973 | 220 | 48.25 |
| Brasov | BV | 630,807 | 927 | 146.95 |
| Braila | BR | 356,196 | 213 | 59.80 |
| Bucharest | B | 2,106,144 | 677 | 32.14 |
| Buzau | BZ | 478,811 | 496 | 103.59 |
| Calarasi | CL | 317,293 | 543 | 171.14 |
| Caras-Severin | CS | 328,047 | 1113 | 339.28 |
| Cluj | CJ | 721,955 | 338 | 46.82 |
| Constanta | CT | 769,768 | 437 | 56.77 |
| Covasna | CV | 228,732 | 72 | 31.48 |
| Dimbovita | DB | 528,426 | 224 | 42.39 |
| Dolj | DJ | 700,117 | 527 | 75.27 |
| Galati | GL | 631,669 | 175 | 27.70 |
| Giurgiu | GR | 276,781 | 126 | 45.52 |
| Gorj | GJ | 366,261 | 158 | 43.14 |
| Harghita | HR | 333,674 | 280 | 83.91 |
| Hunedoara | HD | 469,853 | 201 | 42.78 |
| Iasi | IS | 919,049 | 578 | 62.89 |
| Ialomita | IL | 293,658 | 258 | 87.86 |
| Ilfov | IF | 390,751 | 141 | 36.08 |
| Maramures | MM | 525,765 | 9 | 1.71 |
| Mehedinti | MH | 286,678 | 119 | 41.51 |
| Mures | MS | 595,948 | 498 | 83.56 |
| Neamt | NT | 577,359 | 388 | 67.20 |
| Olt | OT | 450,094 | 225 | 49.99 |
| Prahova | PH | 809,052 | 473 | 58.46 |
| Salaj | SJ | 247,537 | 100 | 40.40 |
| Satu Mare | SM | 390,639 | 956 | 244.73 |
| Sibiu | SB | 464,202 | 385 | 82.94 |
| Suceava | SV | 743,645 | 164 | 22.05 |
| Teleorman | TR | 389,433 | 178 | 45.71 |
| Timis | TM | 742,886 | 1228 | 165.30 |
| Tulcea | TL | 244,249 | 47 | 19.24 |
| Vilcea | VL | 403,171 | 482 | 119.55 |
| Vaslui | VS | 479,815 | 39 | 8.13 |
| Vrancea | VN | 391,169 | 198 | 50.62 |

**^4^** Data obtained from the National Institute of Statistics. *The population of Romania by localities*. 2016. Available online: http://www.insse.ro/cms/sites/default/files/field/publicatii/populatia_romaniei_pe_localitati_la_1ianuarie2016_0.pdf (accessed on 18 February 2019)

**^5^** Data obtained from the weekly reports on measles (2016 to 2018) released by the Romanian National Centre for the Surveillance and Control of Communicable Diseases (CNSCBT). Available online: <https://cnscbt.ro/index.php/informari-saptamanale/rujeola-1> (accessed on 18 February 2019)
