# Supplementary material for: Measles Epidemics in Romania: Lessons for Public Health and Future Policy
Source: Front Public Health. 2019 Apr 25;7:98. doi: 10.3389/fpubh.2019.00098 (PMC6496956; doi:10.3389/fpubh.2019.00098)
Supplement: Supplementary Table 3 — The evolution of the 2016 measles epidemic in Romania. [file Table_3.DOCX]

**Supplementary Table 3:** *The evolution of the 2016 measles epidemic in Romania.*

| **2016** | |  | **2017** | |  | **2018** | |
| --- | --- | --- | --- | --- | --- | --- | --- |
| **Date** | **New Cases** |  | **Date** | **New Cases** |  | **Date** | **New Cases** |
| 30 Sep | 55 |  | 06 Jan | 46 |  | 05 Jan | 28 |
| 07 Oct | 23 |  | 13 Jan | 150 |  | 12 Jan | 116 |
| 14 Oct | 33 |  | 20 Jan | 154 |  | 19 Jan | 86 |
| 28 Oct | 149 |  | 27 Jan | 38 |  | 26 Jan | 50 |
| 11 Nov | 257 |  | 03 Feb | 76 |  | 02 Feb | 64 |
| 18 Nov | 116 |  | 10 Feb | 234 |  | 09 Feb | 126 |
| 25 Nov | 58 |  | 17 Feb | 404 |  | 16 Feb | 87 |
| 09 Dec | 123 |  | 24 Feb | 38 |  | 23 Feb | 77 |
| 16 Dec | 236 |  | 03 Mar | 87 |  | 02 Mar | 123 |
| 22 Dec | 185 |  | 10 Mar | 250 |  | 09 Mar | 87 |
| 29 Dec | 59 |  | 17 Mar | 353 |  | 16 Mar | 189 |
|  |  |  | 24 Mar | 112 |  | 23 Mar | 257 |
|  |  |  | 31 Mar | 114 |  | 30 Mar | 215 |
|  |  |  | 07 Apr | 65 |  | 06 Apr | 204 |
|  |  |  | 14 Apr | 703 |  | 13 Apr | 199 |
|  |  |  | 21 Apr | 88 |  | 20 Apr | 181 |
|  |  |  | 28 Apr | 238 |  | 27 Apr | 280 |
|  |  |  | 05 May | 171 |  | 04 May | 151 |
|  |  |  | 12 May | 438 |  | 11 May | 192 |
|  |  |  | 19 May | 396 |  | 18 May | 335 |
|  |  |  | 26 May | 310 |  | 25 May | 237 |
|  |  |  | 06 Jun | 182 |  | 04 Jun | 199 |
|  |  |  | 09 Jun | 127 |  | 08 Jun | 109 |
|  |  |  | 16 Jun | 490 |  | 15 Jun | 271 |
|  |  |  | 23 Jun | 49 |  | 22 Jun | 104 |
|  |  |  | 30 Jun | 208 |  | 29 Jun | 142 |
|  |  |  | 07 Jul | 157 |  | 06 Jul | 208 |
|  |  |  | 14 Jul | 370 |  | 13 Jul | 105 |
|  |  |  | 21 Jul | 229 |  | 20 Jul | 57 |
|  |  |  | 28 Jul | 101 |  | 27 Jul | 67 |
|  |  |  | 04 Aug | 108 |  | 03 Aug | 29 |
|  |  |  | 11 Aug | 38 |  | 09 Aug | 70 |
|  |  |  | 18 Aug | 310 |  | 24 Aug | 103 |
|  |  |  | 25 Aug | 134 |  | 31 Aug | 82 |
|  |  |  | 01 Sep | 45 |  | 07 Sep | 78 |
|  |  |  | 08 Sep | 123 |  | 14 Sep | 80 |
|  |  |  | 15 Sep | 128 |  | 21 Sep | 85 |
|  |  |  | 22 Sep | 56 |  | 28 Sep | 15 |
|  |  |  | 29 Sep | 132 |  | 05 Oct | 12 |
|  |  |  | 06 Oct | 118 |  | 12 Oct | 16 |
|  |  |  | 13 Oct | 87 |  | 19 Oct | 13 |
|  |  |  | 20 Oct | 44 |  | 26 Oct | 26 |
|  |  |  | 27 Oct | 34 |  | 02 Nov | 12 |
|  |  |  | 03 Nov | 24 |  | 16 Nov | 24 |
|  |  |  | 10 Nov | 94 |  | 23 Nov | 23 |
|  |  |  | 17 Nov | 78 |  | 30 Nov | 26 |
|  |  |  | 24 Nov | 46 |  | 07 Dec | 21 |
|  |  |  | 01 Dec | 64 |  | 14 Dec | 15 |
|  |  |  | 08 Dec | 106 |  | 21 Dec | 32 |
|  |  |  | 15 Dec | 82 |  |  |  |
|  |  |  | 22 Dec | 70 |  |  |  |
|  |  |  | 29 Dec | 11 |  |  |  |

Data obtained from the weekly reports on measles (2016 to 2018) released by the Romanian National Centre for the Surveillance and Control of Communicable Diseases (CNSCBT). Available online: <https://cnscbt.ro/index.php/informari-saptamanale/rujeola-1> (accessed on 18 February 2019)
